# Corrections to the following abstracts

**DOI:** 10.1093/bib/bbag080

**Published:** 2026-02-10

These are corrections to:

Hideki Tanizawa, Claire Yik-Lok Chung, Ken-ichi Noma, Integrative analysis of hi-C variance and multi-contact data identifies enhancer–promoter hub structures, *Briefings in Bioinformatics*, Volume 26, Issue Supplement_1, December 2025, Page i27, https://doi.org/10.1093/bib/bbaf631.039

and

Claire Yik-Lok Chung, Shinya Ohta, Hideki Tanizawa, Ken-ichi Noma, DeepSAHF: deep learning-based detection of senescence-associated chromatin features reveals nuclear heterogeneity, *Briefings in Bioinformatics*, Volume 26, Issue Supplement_1, December 2025, Page i27, https://doi.org/10.1093/bib/bbaf631.040

In both papers, a co-author's first and last names are emended to be separated instead of erroneously joined, and now read: “Hideki Tanizawa”, and are present in both PDF and digital versions of the paper.

